# The tammar wallaby: A marsupial model to examine the timed delivery and role of bioactives in milk

**DOI:** 10.1016/j.ygcen.2016.08.007

**Published:** 2017-04-01

**Authors:** Julie A. Sharp, Stephen Wanyonyi, Vengama Modepalli, Ashalyn Watt, Sanjana Kuruppath, Lyn A. Hinds, Amit Kumar, Helen E. Abud, Christophe Lefevre, Kevin R. Nicholas

**Affiliations:** a Institute for Frontier Materials, Deakin University, Geelong 3216, Australia; b School of Medicine, Deakin University, Geelong 3216, Australia; c Cancer Program, Monash Biomedicine Discovery Institute and Department of Anatomy and Developmental Biology, Monash University, Clayton 3800, Victoria, Australia; d CSIRO Health and Biosecurity, Canberra, ACT 2601, Australia; e Division of Bioinformatics, Walter and Eliza Hall Medical Research Institute, Melbourne, Victoria 3000, Australia; f PeterMac Callum Cancer Research Institute, East Melbourne 3002, Victoria, Australia; g Department of Medical Biology (WEHI), The University of Melbourne, Melbourne 3000, Victoria, Australia; h Institute for Agriculture and the Environment, University of Southern Queensland, Toowoomba, QLD 4350, Australia

**Keywords:** Lactation, Milk, Mammary gland, Marsupial, Monotreme

## Abstract

It is now clear that milk has multiple functions; it provides the most appropriate nutrition for growth of the newborn, it delivers a range of bioactives with the potential to stimulate development of the young, it has the capacity to remodel the mammary gland (stimulate growth or signal cell death) and finally milk can provide protection from infection and inflammation when the mammary gland is susceptible to these challenges. There is increasing evidence to support studies using an Australian marsupial, the tammar wallaby (*Macropus eugenii*), as an interesting and unique model to study milk bioactives. Reproduction in the tammar wallaby is characterized by a short gestation, birth of immature young and a long lactation. All the major milk constituents change substantially and progressively during lactation and these changes have been shown to regulate growth and development of the tammar pouch young and to have roles in mammary gland biology. This review will focus on recent reports examining the control of lactation in the tammar wallaby and the timed delivery of milk bioactivity.

## 1 Introduction

Lactation evolved about 200 million years ago in aplacental egg laying animals as observed today in monotremes (class Mammalia, subclass Prototheria) but since this time there has been extensive adaptation to reproduction, including a large variation of lactational strategies when the Theria split into the Metatheria (Marsupialia) and Eutheria (Placentalia) lineages over 150 Mya (Lefevre et al., 2010).

Many of these species have extreme adaption to lactation and the availability of comparative functional genomics now show that the regulatory mechanisms controlling the great majority of physiological processes have been conserved during evolution but the timing and mechanism for delivering these processes may differ between species of mammals (Sharp et al., 2014). Therefore the use of these diverse species coupled with the availability of genomics and bioinformatics has provided the opportunity to exploit marsupial models for new insights into the functions of milk.

Eutherians have a well-developed placenta and a long gestation that leads to the birth of a relatively well developed young. The length of lactation is often similar to gestation and composition of the milk doesn’t change substantially. In contrast, the echidna (*Tachyglossus* and *Zaglossus* genera) and platypus (*Ornithorhynchus anatinus*; monotremes), and the tammar wallaby (*Macropus eugenii*; a marsupial) are increasingly studied groups of animals (Sharp et al., 2015, Sharp et al., 2014). The echidna has an interesting combination of reptilian and mammalian characters. It has retained a primitive component of reptilian reproduction, laying shelled eggs (Morrow and Nicol, 2013) but subsequently milk is the sole source of nutrition and protection for the hatchlings which are altricial and are not immune competent (Griffiths, 1978). The early stages of development of this altricial young occurs in a non-sterile environment therefore the role of milk is not only important for growth and development but also particularly essential for protection of the young from disease (Bisana et al., 2013, Enjapoori et al., 2014). It has been postulated that antibacterial bioactives fulfilling a role for the protection and survival of the young have been integral to the evolution of mammals (Sharp et al., 2011). Reproduction in marsupials such as the tammar wallaby (*M. eugenii*) is characterized by a short gestation (26.5 days), birth of immature young and a long lactation (approximately 300 days) during which all the major milk constituents change substantially during lactation (Tyndale-Biscoe and Janssens, 1988). Interestingly the tammar neonate closely resembles a fetus and remains attached to the teat in the pouch for the first 100 days of lactation, and may be considered a fetus maintained in the pouch as opposed to the uterus. Indeed, the efficiency of conversion of milk to body mass is very similar to the conversion of precursors to body mass observed in the eutherian fetus *in utero* (Tyndale-Biscoe and Renfree, 1987).

**Fig. 1:**
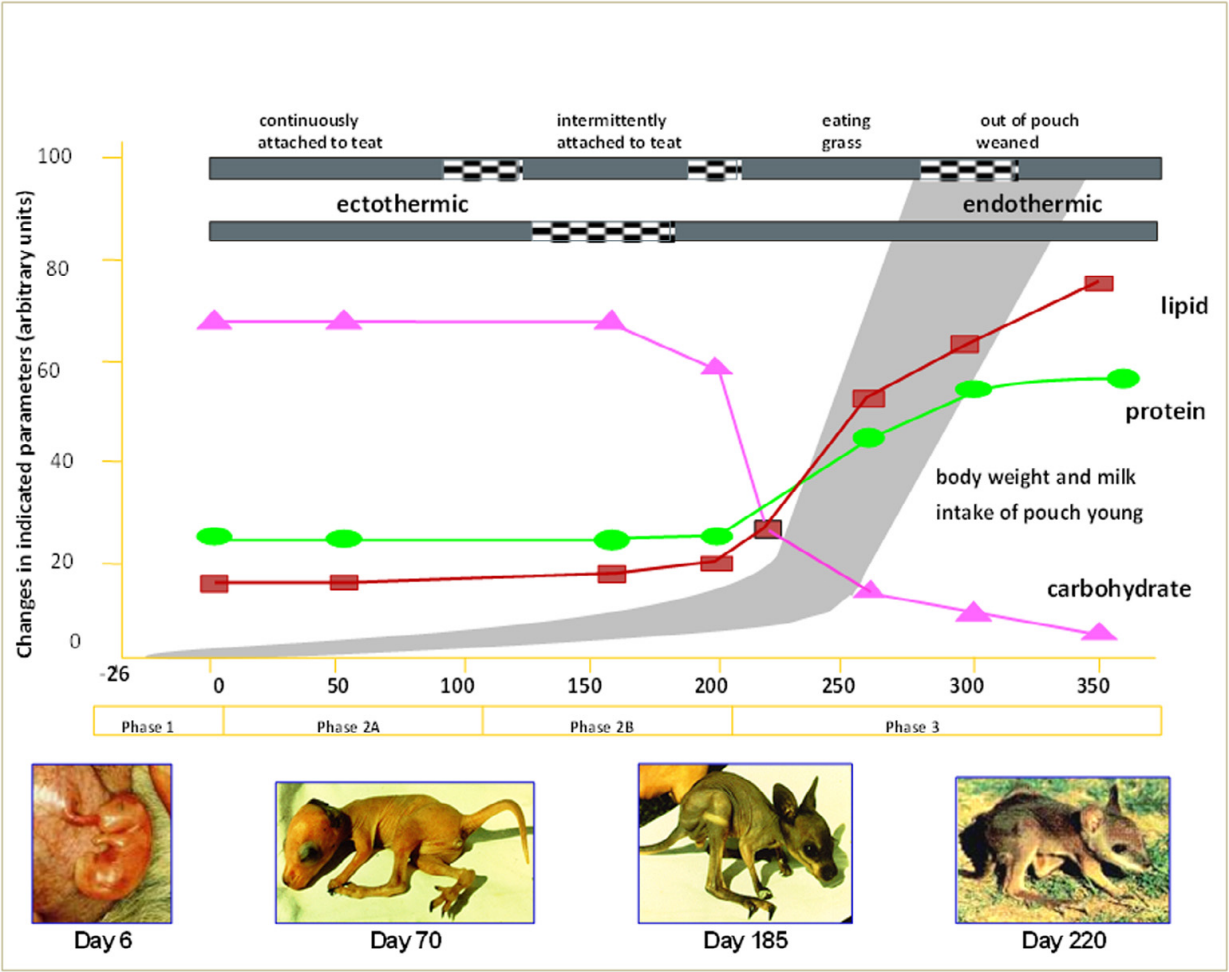
Tammar wallaby lactation strategy. Progressive changes in milk production, milk composition and growth of the young during the three phases of the lactation cycle in the tammar wallaby (Brennan et al., 2007a, Nicholas et al., 1997).

There is increasing evidence that changes in milk composition regulate growth of the tammar pouch young (Sharp et al., 2010, Sharp et al., 2015). The tammar wallaby is one of most studied marsupials and its lactation is divided into three phases (phase 2A, phase 2B and phase 3) based on the composition of the milk and growth and development of the young (Fig. 1) (Tyndale-Biscoe and Janssens, 1988). During the first 100 days the development of the marsupial neonate is similar to a late stage eutherian fetus and therefore the signalling factors involved in the development of the eutherian fetus are most likely delivered in the milk (Brennan et al., 2007a). The pouch young are born with immature organs and during early lactation the organs necessary for their survival such as respiratory system (Runciman et al., 1996), gut (Kwek et al., 2009a), lymphoid tissues (Basden et al., 1997) and nervous system including brain and spinal-cord (Harrison and Porter, 1992, Saunders et al., 1989) are rapidly developed. Fostering experiments demonstrated that transferring the early phase pouch young to a late phase lactating tammar can accelerate the growth and physical development of pouch young (Menzies et al., 2007, Trott et al., 2003). Subsequent studies showed that cross fostering the young also accelerated maturation of specific organs such as the stomach (Kwek et al., 2009a). More recent studies have shown that the lungs of the marsupial neonate are immature and develop rapidly in the suckled young during the early stage of lactation (Modepalli et al., 2015). Using *in vitro* models it was shown that milk collected from marsupials during early lactation (day 20–100), but not late lactation (day 100–300) stimulated proliferation and differentiation of whole lung cultures from mouse embryos (Modepalli et al., 2015). These studies are still preliminary in terms of identifying the factors that have the potential to stimulate lung development but the models allow for a focused examination of milk between day 20 and 100 of lactation to identify candidate signalling molecules.

Interestingly, the tammar can practise concurrent asynchronous lactation (Nicholas, 1988a, Nicholas, 1988b); the mother provides a concentrated milk for an older animal which is out of the pouch and a dilute milk from an adjacent mammary gland for a newborn pouch young, suggesting the mammary gland is most likely under the control of both endocrine and local regulatory mechanisms. An explanation for the mechanisms controlling this phenomenon has eluded scientists for more than 80 years but more recent studies are starting to shed new light on the central role of the extracellular matrix in programming the mammary epithelial cells to produce milk with a specific composition (Wanyonyi et al., 2013). This data will be important to better understand how the tammar regulates a timed delivery of bioactives in milk.

This review will focus on the more recent studies examining the control of lactation in the tammar wallaby and the timed delivery of milk bioactivity. As mentioned above, the major growth and development of the young occurs *post-partum* during lactation and therefore the immature marsupial neonates rely on maturation factors in milk that would most likely be presented to the eutherian fetus by the placenta and amniotic fluid (Nicholas et al., 1997). There is a high incidence of premature and low birthweight (LBW) human babies in developing countries (Lee et al., 2013). This leads to either inappropriate or lack of signalling of organ development which results in a failure to thrive (initially a result of limited gut and lung development), higher incidence of death and increased frequency of mature onset disease (eg hypertension, diabetes, obesity). Studying the milk from Australian marsupials provides a unique opportunity to identify factors that program short and long-term development of the young; the human orthologue of these proteins may hold considerable promise as a supplement for improved short and long-term health outcomes for LBW and premature babies (both breast and formula fed).

## 2 The lactation cycle in the tammar wallaby

The tammar mammary gland is programmed to progress through defined phases of the lactation cycle and to deliver milk bioactivity that correlates with specific developmental stages in the young (Sharp et al., 2010). The tammar lactation cycle is divided into four broad phases (Fig. 1). The gestation phase (P1) is approximately 26 days with the subsequent birth of a fetus-like young (Tyndale-Biscoe and Renfree, 1987). During P1 all the four mammary glands undergo progressive lobulo-alveolar development, gradually replacing the connective tissue with glandular tissue (Findlay, 1982). Phase 2A (P2A) commences at parturition when the neonate attaches permanently to one of the four teats and remains attached for approximately 100 days (Behringer et al., 2006, Brennan et al., 2007b, Nicholas, 1988a, Old and Deane, 2000, Tyndale-Biscoe and Renfree, 1987). During this early stage of development the young is altricial and immunocompromised with a limited capacity to mount an immune response (Basden et al., 1997). The mother produces relatively small volumes of dilute milk with a high concentration of complex carbohydrates and a low concentration of protein and lipid (Hendry et al., 1998, Nicholas et al., 2001, Trott et al., 2003) (Fig. 1). The reproductive strategy of the mother is to slow growth of the pouch young while the tissues develop and become functional. Phase 2B (P2B) commences 100–120 days *post-partum* and continues for approximately 100 days during which the neonate remains in the pouch but relinquishes the teat and only re-attaches to suck (Hendry et al., 1998, Trott et al., 2003). The milk produced maintains high levels of carbohydrates and low concentrations of protein and lipids. However, there are changes in the kinds of proteins secreted (Brennan et al., 2007a, Nicholas et al., 1995).

At the onset of phase 3 (P3), the neonate begins to exit the pouch and feeds on herbage, returning to the pouch to suckle. During P3 the mammary gland enlarges significantly (Bird et al., 1994), producing large amounts of concentrated milk that is rich in protein and lipid but low in carbohydrates to provide a high energy milk (Green and Merchant, 1988, Hendry et al., 1998, Nicholas et al., 1997, Trott et al., 2003, Tyndale-Biscoe and Renfree, 1987). P3 also represents the period of most dramatic change in morphology and growth of the young including the switch from ectothermic to endothermic regulation of body temperature (Janssens et al., 1997, Nicholas et al., 1997, Nicholas, 1988a, Richardson et al., 2002).

**Fig. 2:**
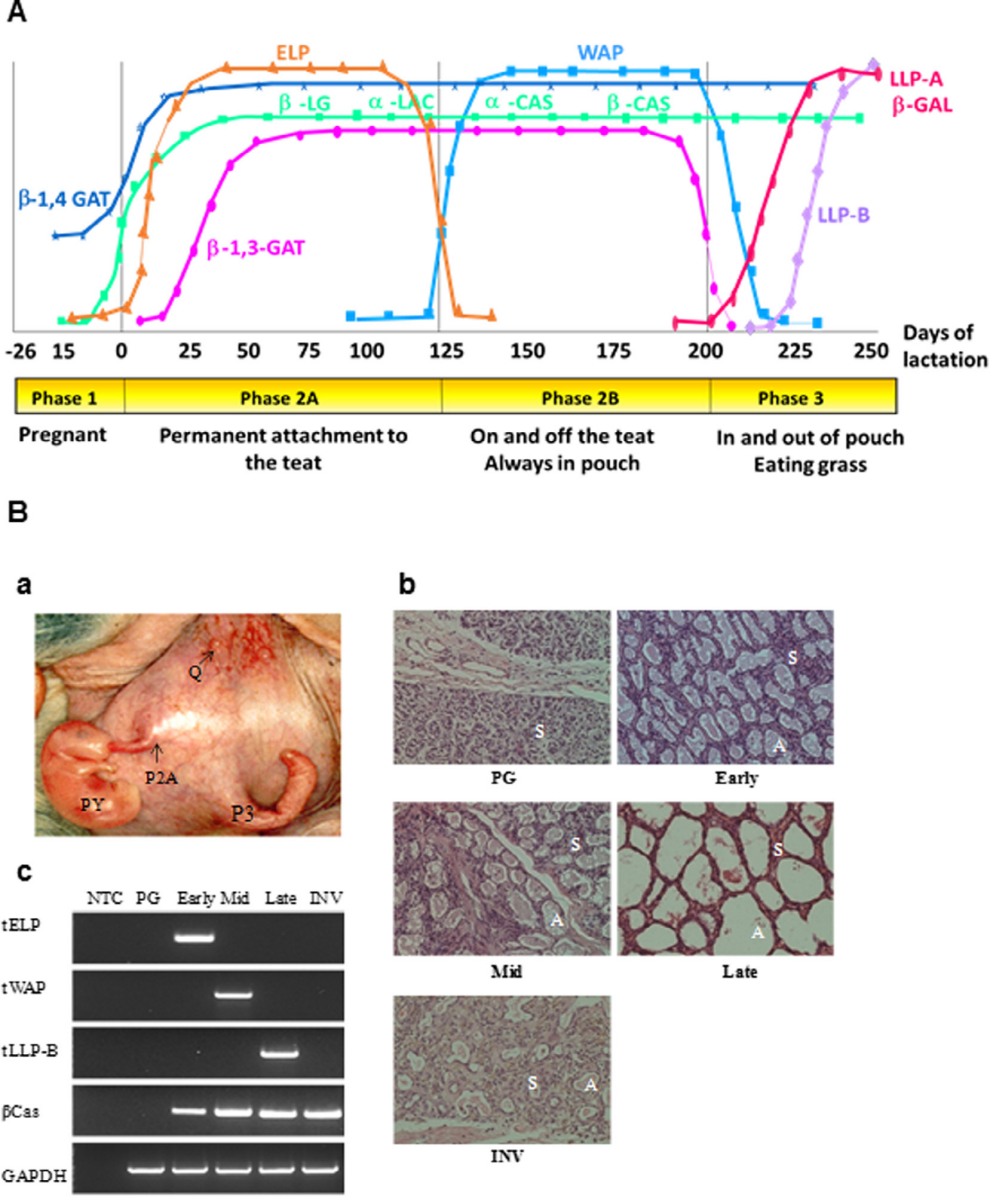
(A) Differential expression of the major milk protein genes during tammar lactation. Profile of tammar milk protein genes expressed during the lactation cycle. The genes shown are α-casein (α-CAS), β-casein (β-CAS), α-lactalbumin (α-LAC), β-lactoglobulin (β-LG, early lactation protein (ELP), whey acidic protein (WAP), late lactation protein-A (LLP-A) and late lactation protein-B (LLP-B). Adapted from Sharp et al. (2011). (B) Phase specific mammary morphology and milk protein gene expression. (a) Tammar mammary glands showing asynchronous lactation. Q represents quiescent and non-lactating mammary gland, P2A and P3 represent mammary glands producing early lactation milk for the pouch young (PY) and late lactation milk for the young that has exited the pouch respectively. (b) H&E stained cross sections of mammary gland. PG (pregnant, day 17), Early (early lactation, day 40), Mid (mid-lactation, day 168), Late (late lactation, day 260) and INV (involution, day 10 post weaning). Alveoli are marked with A, stroma with S. (c) RT-PCR analysis of milk protein gene expression. NTC, no template control. Stages of lactation as above. tELP, tWAP, tLLP-B and tβ-cas represent tammar early lactation protein, whey acidic protein, late lactation protein-B and β-casein, respectively. GAPDH was used as the housekeeping gene. PCR products were resolved on a 1.2% agarose gel. Adapted from (Nicholas et al., 2012).

Microarray analysis of the tammar mammary gland has revealed a multitude of changes in gene expression during the lactation cycle (Brennan et al., 2007a, Sharp et al., 2009, Sharp et al., 2015) but earlier studies have identified some of the major milk protein genes that can be used as markers to identify the specific phases of milk production (Menzies and Nicholas, 2007, Nicholas et al., 1997, Nicholas, 1988a, Trott et al., 2003, Trott et al., 2002) (Fig. 2A). P2A is characterized by the expression of the gene for the early lactation protein (tELP) (Khalil et al., 2008, Simpson et al., 1998a, Trott et al., 2003), P2B by the expression of the gene for the whey acidic protein (tWAP) (Nicholas et al., 2001, Simpson et al., 2000) and P3 by the expression of the gene for the Late lactation proteins (LLP-A and LLP-B) (Trott et al., 2003, Trott et al., 2002). In contrast to these asynchronously expressed genes, the caseins, β-lactoglobulin and α-lactalbumin genes are expressed throughout lactation (Sharp et al., 2009) which is similar to observations in eutherian mammals.

In the early 1960s asynchronous concurrent lactation (ACL) was described in macropod marsupials (Griffiths et al., 1972, Nicholas, 1988a) with the marsupial having a dormant blastocyst in the uterus and at the same time two parallel lactation cycles producing early and late lactation milk in adjacent glands to support a pouch young and an older sibling at heel concurrently (Fig. 2B). Subsequent experiments showed that apart from the different volumes of milk secreted from adjacent teats, there was differential expression of specific milk protein genes in each mammary gland, suggesting a local intra-mammary mechanism for ACL (Bird et al., 1994, Brennan et al., 2007b, Nicholas et al., 1997). It is likely that paracrine and potentially autocrine factors, in addition to endocrine factors modulate ACL since the observed differences between the individual mammary glands occurs in the presence of the same hormonal milieu. Therefore, the tammar provides a challenging experimental model to understand the concomitant regulatory mechanisms of the lactation cycle but more specifically this new data will provide a better understanding of the mechanisms of local regulation of milk composition.

## 3 Endocrine control of the tammar mammary gland

Previous studies using a tammar mammary explant culture model have shown that mammary tissue from pregnant (P1) tammars incubated with different combinations of insulin, cortisol, prolactin and thyroid hormone lead to expression of the individual casein genes and whey protein genes such as β-lactoglobulin and α-lactalbumin that are normally expressed in P2A mammary gland (Simpson and Nicholas, 2002, Simpson et al., 2000). In contrast, the LLP genes could be down-regulated in mammary explants from P3 tammars and then restimulated with insulin, cortisol, and prolactin, but expression of these genes could not be induced in mammary explants from pregnant tammars with any hormone combination tested (Trott et al., 2002). This conclusion was supported by experiments showing that constructs comprised of the *Llp-A* gene promoter and a reporter gene did not express after transfection into Chinese hamster ovary cells incubated with insulin, cortisol, and prolactin. The same construct was not expressed at any stage of the lactation cycle in transgenic mice (Trott et al., 2002). Therefore it was evident that some of the genes expressed by the tammar were regulated by multiple mechanisms and that alternative models were required to better understand these processes.

## 4 The mammary extracellular matrix – a role in differential expression of tammar milk protein genes

Mammary epithelial cells (MEC) are attached to extracellular matrix (ECM) which transduces signals necessary for modulation of histogenic processes including apical/basal cell polarity (Maller et al., 2010, Schedin et al., 2004) and cell proliferation (Provenzano et al., 2009). Therefore, the local regulation of mammary function in adjacent glands during ACL suggests that, in addition to endocrine stimuli, the ECM in individual glands may have a role in determining which genes are expressed in the mammary epithelial cells.

The concept of ACL in macropod marsupials has remained perplexing because two adjacent mammary glands under the same systemic control not only differ significantly in size but produce milk with profoundly different composition (Lincoln and Renfree, 1981, Nicholas et al., 1997, Nicholas, 1988a). Several mechanisms have been suggested for ACL (Lincoln and Renfree, 1981, Nicholas et al., 1997, Nicholas and Tyndale-Biscoe, 1985) but in a recent study by Wanyonyi et al. (2013), tammar mammary epithelial cells (WallMECs) and ECM collected from mammary tissue at different phases of lactation were used in a series of experiments where the cells from one phase were cultured on ECM from another phase to investigate ECM-induced changes in marker genes.

Initial experiments showed that the ECM regulated the morphology of the 3-dimensional alveolar-like acini which are formed by the wallaby mammary epithelial cells (WallMEC) cultured on mammary ECM. WallMECs from mid lactation cultured on ECM extracted from late lactation mammary glands developed acini that were more numerous and smaller in size than acini cultured on ECM extracted from mid lactation mammary glands (Wanyonyi et al., 2013). These data provided the first indication that phase-specific ECM influenced WallMECs to alter the morphology of mammary acini and led to subsequent experiments using this model to assess the impact of ECM on milk protein gene expression in WallMECs.

## 5 Late phase mammary ECM changes the phenotype of earlier phase WallMEC to resemble the latter phase of lactation

**Fig. 3:**
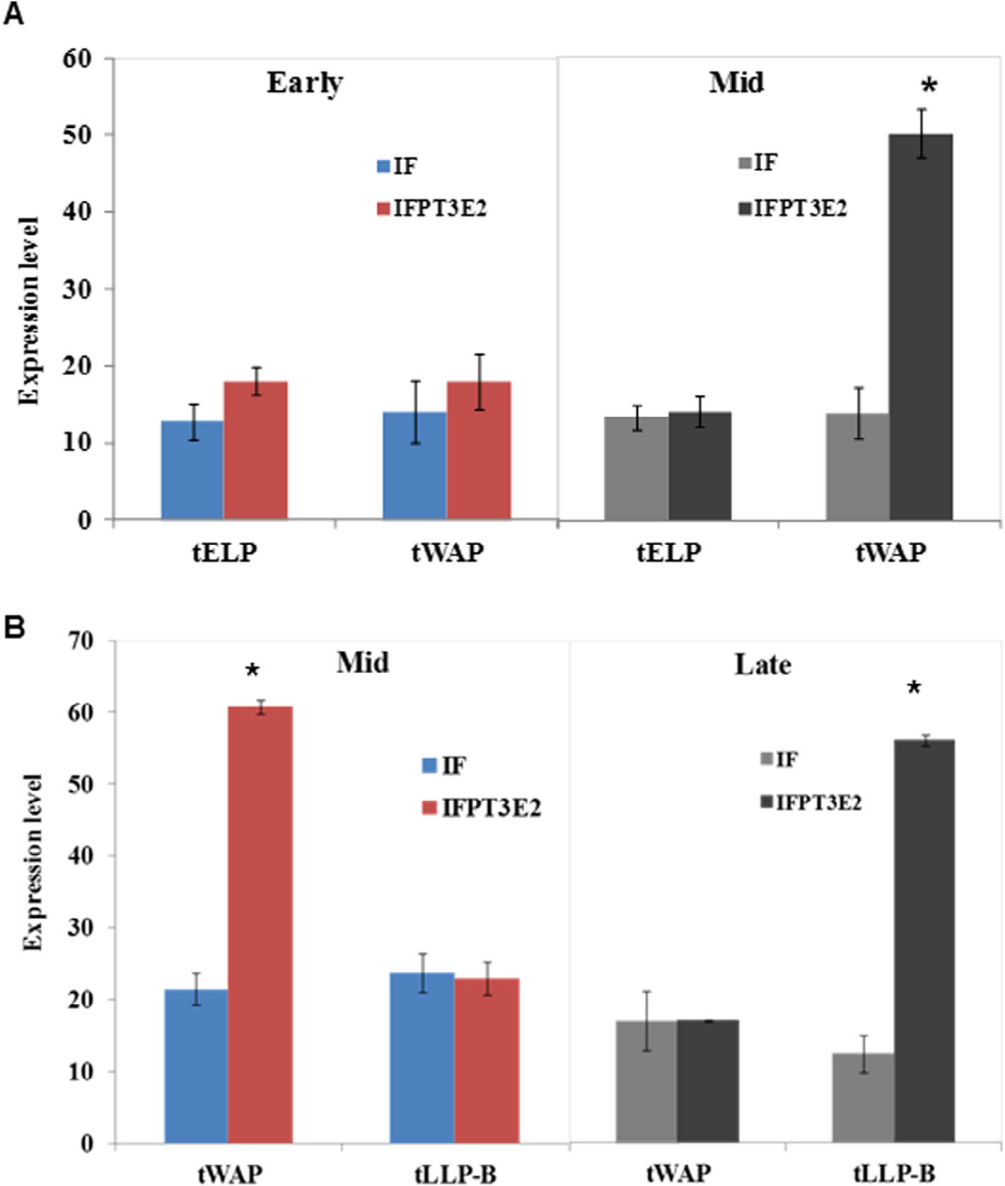
Milk protein gene expression after culturing early and mid lactation WallMECs on mammary ECM. (A) tELP and tWAP gene expression in WallMEC cultured on early and mid lactation mammary ECM. Gene expression was analysed relative to GAPDH expression. Early and Mid represent the lactation phase of the ECM. Samples were either treated with either insulin and cortisol (IF) or insulin, cortisol, prolactin, tri-iodothyronine and estradiol (IFPT_3_E_2_). The asterisk denotes significant difference (Student *t*-test, p = 0.011; n = 4) between early and mid lactation Standard error bars (SEM) are shown. (B) tWAP and LLP-B gene expression in WallMEC cultured on mid and late lactation mammary ECM. Mid and Late represent the lactation phase of the ECM. The asterisk represents significant difference (p ⩽ 0.005; n = 4) between mid and late lactation ECM. Adapted from (Wanyonyi et al., 2013).

When early lactation cells were cultured on mid lactation ECM they expressed significantly higher levels of the *tWAP* gene compared to when they were cultured on early lactation ECM (Fig. 3A). Similarly when mid lactation cells were cultured on late lactation ECM they expressed significantly more *tLLP-B* (Fig. 3B) but less *tWAP* than when they were cultured on their own phase (mid) ECM. In all the treatments the expression of milk protein genes in MECs was dependent on the inclusion of I, F and P in culture media which is consistent with the requirement for these hormones for milk protein gene expression in mammary explants (Wanyonyi et al., 2013).

## 6 Earlier phase mammary ECM does not reverse the phenotype of latter phase WallMEC to the earlier phase of lactation

**Fig. 4:**
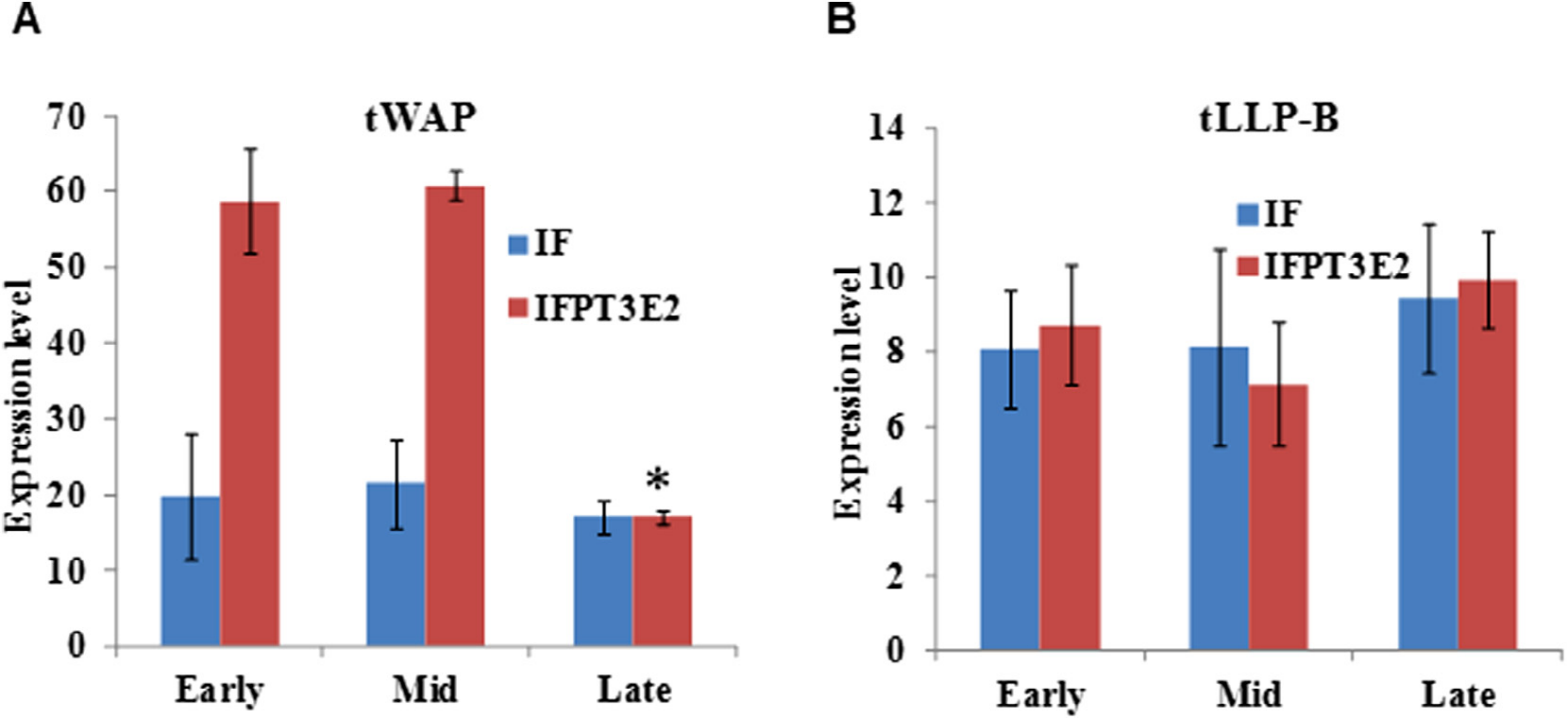
Effect of ECM on milk protein gene expression (A) tWAP gene expression in early lactation WallMEC cultured on early, mid and late lactation mammary ECM. Gene expression was analysed relative to GAPDH. Early, Mid and Late represent the lactation phase of the ECM. The asterisk denotes significantly lower expression compared to mid lactation ECM. Standard error bars (SEM) are shown. Statistical analysis was performed using Student *t*-test (p < 0.005; n = 4). (B) LLP-B gene expression in early lactation WallMEC cultured on early, mid and late lactation ECM. Adapted from (Wanyonyi et al., 2013).

WallMEC from a late phase of lactation were cultured on ECM from an earlier phase to determine if this treatment would reverse milk protein gene expression to the earlier phase phenotype. To test this hypothesis mid lactation cells were cultured on early, mid and late lactation ECM and the expression of the *tWAP* gene measured (Wanyonyi et al., 2013). There was no difference in expression of *tWAP* between cells cultured on early and mid lactation ECM but *tWAP* expression was significantly lower on late lactation ECM (Fig. 4A). This suggested that the WallMECs were developmentally programmed in a forward direction that could not be reversed.

In order to confirm whether the ECM changes the phase phenotype of WallMEC in a sequential manner, early lactation cells were cultured on early, mid and late lactation ECM and the expression of *tLLP-B* measured. There was no significant difference in *tLLP-B* gene expression between early lactation cells cultured on mid or late lactation ECM and their own phase (early lactation) ECM (Wanyonyi et al., 2013). These experiments suggested that the WallMECs must be sequentially programmed at each phase of the lactation cycle before proceeding to the next phase of lactation.

Wanyonyi et al. (2013) demonstrated that the transition between phases appeared to correlate with the progressive changes in ECM composition of the mammary gland across the lactation cycle. However, an important experiment would be to culture early lactation WallMECs on P2B ECM and then transfer the cells to a P3 ECM to examine whether there is a need to progressively program the cells on each phase ECM to enable the cells to eventually express P3 milk protein genes.

In summary, the regulation of mammary development and mammary function during the lactation cycle is underpinned by a sophisticated program of control that requires signals from the endocrine system and the extracellular matrix. This program requires that the MECs proceed through a development program that continues in one direction from P1 to P3 of lactation and cells must systematically progress through each phase of lactation in order to advance to the next phase. This multilevel control of milk composition is necessary to provide both timed delivery of bioactivity and protection from infection to the developing young and potentially to assist in the regulation of mammary development.

## 7 The role of milk bioactives in development of the suckled young

### 7.1 Development of the gut

Dramatic changes in gut morphology in the suckled young take place around day 170 *post-partum* (Waite et al., 2005). In the hindstomach region, parietal cells increase in number, gastric glands enlarge and adopt the adult-like phenotype of very long, thin glands (Waite et al., 2005) and peptic enzyme activity becomes elevated (Davis, 1981). Concomitantly the forestomach region changes from an immature gastric glandular phenotype to a cardia glandular phenotype in the region that will become the adult forestomach (Kwek et al., 2009a, Waite et al., 2005). The phenotypic change in the forestomach was accompanied by functional changes; an increase in pH to neutrality (Janssens and Ternouth, 1987), a decline in peptic activity (Davis, 1981) and the gastric glandular cell type gene markers were down-regulated (Kwek et al., 2009a). The changes in stomach morphology were correlated with significant changes in milk composition raising the possibility that these processes may be regulated by milk bioactives. Indeed, a study by Kwek et al., 2009a, Kwek et al., 2009b examined pouch young (PY) at day 120 of age cross-fostered to host mothers at day 170 of lactation for 50 days. Analysis of the fore-stomach in fostered PY showed there was increased apoptosis, but no change in cell proliferation (Kwek et al., 2009b). The parietal cell population was significantly reduced, suggesting that fore-stomach maturation proceeds by two temporally distinct processes that were uncoupled: down-regulation of gastric glandular phenotype and initiation of cardia glandular phenotype. These experiments also indicated that herbage consumed by the PY and bacterial colonisation of the stomach may play additional roles in regulating these two processes.

More recent studies (Kuruppath, Sharp, Nicholas and Abud, unpublished) have shown that milk collected from tammars in early lactation and cultured with embryonic mouse stomach explants resulted in elevated cell proliferation and increased level of expression of specific developmental gene markers. It is likely that these kinds of factors would be delivered by the placenta and amniotic fluid to eutherian fetus. Therefore, identification of these developmental signalling molecules will show promise for new strategies to address limited gut development in premature and low birth weight babies.

### 7.2 Development of the lung

The stages of lung development in mammals are similar (Tschanz, 2007). In eutherians, the majority of lung morphogenesis occurs during gestation, while the placenta performs gaseous exchange between the fetus and the mother (Mess and Ferner, 2010). During this period the lung develops to maturity to enable gaseous exchange after birth. In contrast, studies of lung development in several marsupial species including bandicoot (*Isoodon macrourus*) (Gemmell and Little, 1982), Julia Creek dunnart (*Sminthopsis douglasi*) (Frappell and Mortola, 2000), tammar wallaby and gray short tailed opossum (*Monodelphis domestica*) (Szdzuy et al., 2008) have demonstrated that the major developmental changes in the respiratory system occur during their early postnatal life (Runciman et al., 1996). Unlike eutherians, marsupials allow transmission of macromolecules across the gut wall due to their immature gut (Yadav, 1971) and therefore the milk proteins and peptides transmitted from the intestinal lumen into the peripheral circulation may play a regulatory role in lung maturation that allows a transition from respiration through the skin to gaseous exchange in the lung.

**Fig. 5:**
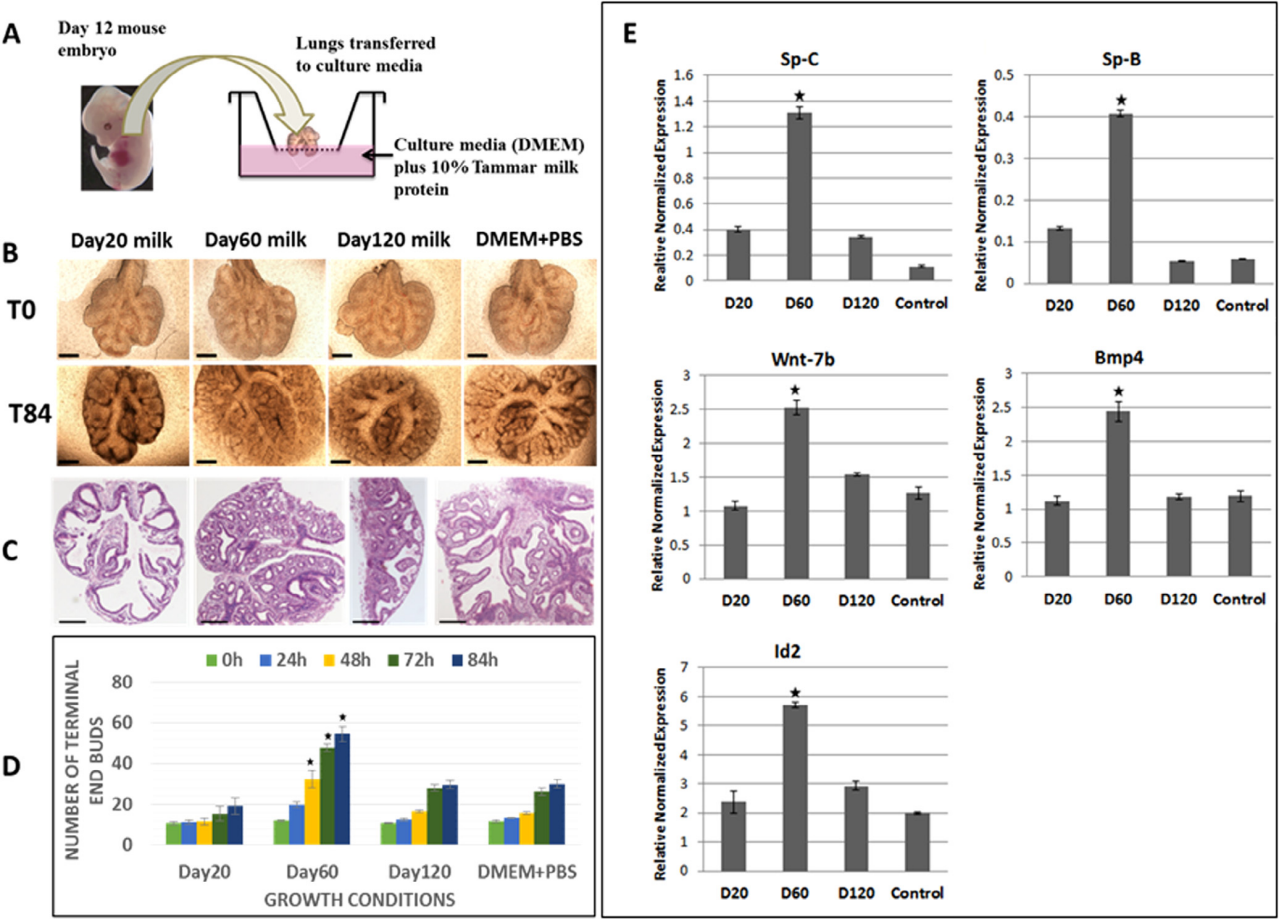
Effect of early phase tammar wallaby milk on lung branching morphogenesis and expression of lung developmental marker genes. (A) The experimental model used for culture of mouse E12 embryonic lungs with tammar milk. (B) Embryonic lungs were cultured for 86 h in the presence of wallaby milk (10%) collected at day 20, day 60, day 120 and control embryonic lungs were cultured in media with PBS. (C) Histological analysis of mouse embryonic lung sections stained with H&E after 84 h of culture. The experimental conditions are described above. (D) Analysis of branching morphogenesis. The number of terminal end buds were counted in sections from control embryonic lung and embryonic lungs treated with tammar milk from day 20, day 60, day 120 lactation. (E) Expression of critical developmental marker genes Sp-C, Sp-B (Type-II pneumocytes marker genes), Wnt-7b, Bmp4 and Id-2 in mouse embryonic lung cultured with tammar milk for 84 h. Significant increases are indicated (P values < 0.05, n = 4) with an asterisk (Student *t*-test). Scale bar 250 μm. Adapted from (Modepalli et al., 2015).

In order to examine the potential role of tammar milk in lung development Modepalli et al. (2015) cultured mouse embryonic lungs (E-12) in media with tammar skim milk collected at key time points during lactation (Fig. 5; Modepalli et al., 2015). Remarkably the embryonic lungs showed increased branching morphogenesis when incubated with milk collected at specific time points between approximately day 40–100 of lactation (P2A) and reduced lung development when incubated in media with milk from day 20 lactation (Fig. 5A–D). In addition, day 60 milk significantly up regulated a number of marker genes for key developmental processes and specialised cell types (Fig. 5E). This suggests that day 20 milk either lacks the necessary factors to stimulate lung development or included inhibitors of this process at a time when respiration occurs through the skin. This appears to be a sophisticated temporal regulation of tissue development in the neonate by milk bioactivity.

The mechanisms by which the day 60 milk stimulated lung development remain to be established but the studies of Modepalli et al. (2015) showed a difference in the ratio of epithelium and mesenchyme in embryonic lung when treated with tammar milk protein collected from different time points. The addition of day 60 milk to mouse embryonic lungs stimulated the primitive mesenchyme, with increased cell proliferation and elongation of mesenchymal cells invading the surrounding matrix. Mesenchymal-epithelial interactions are essential for epithelial branching morphogenesis (Alescio and Cassini, 1962, Masters, 1976) and it was interesting to observe that in media containing day 60 milk proteins the mesenchymal cells were flattened, elongated and spindle shaped, representing either airway smooth muscle cells or myofibroblast cells derived from primary mesenchyme. In contrast, the treatment of mouse embryonic lung with day 20 milk showed a reduced effect on epithelium and mesenchymal cell populations which is consistent with the inhibiting effects of this milk on development of lung explants. This temporal effect was lost in milk collected from later phases of lactation (P2B & P3). Taken collectively the data shows the timing of this stimulatory activity of milk on mouse embryo lungs is consistent with increased lung development in tammar neonates during the first 100 days *post-partum* and the reduced level of lung development after P2A lactation.

## 8 Mechanisms for delivery of bioactivity in milk

### 8.1 Multi-functional milk proteins; domain-specific delivery of bioactivity

It is now becoming clear that alternative splicing of some milk protein genes has been utilised by the mammary gland to deliver domain-specific functions at specific times during lactation.

### 8.2 Cathelicidin (Meucath1)

#### 8.2.1 A role in mammary innate immunity

Cathelicidin includes antimicrobial peptides that form part of innate immunity in vertebrates (Yang et al., 2004) and the protein usually exists in an inactive proform until post-translational cleavage by specific proteases. Earlier studies have shown the two domains of the proform cathelicidin may have a variety of functions (Yang et al., 2004).

**Fig. 6:**
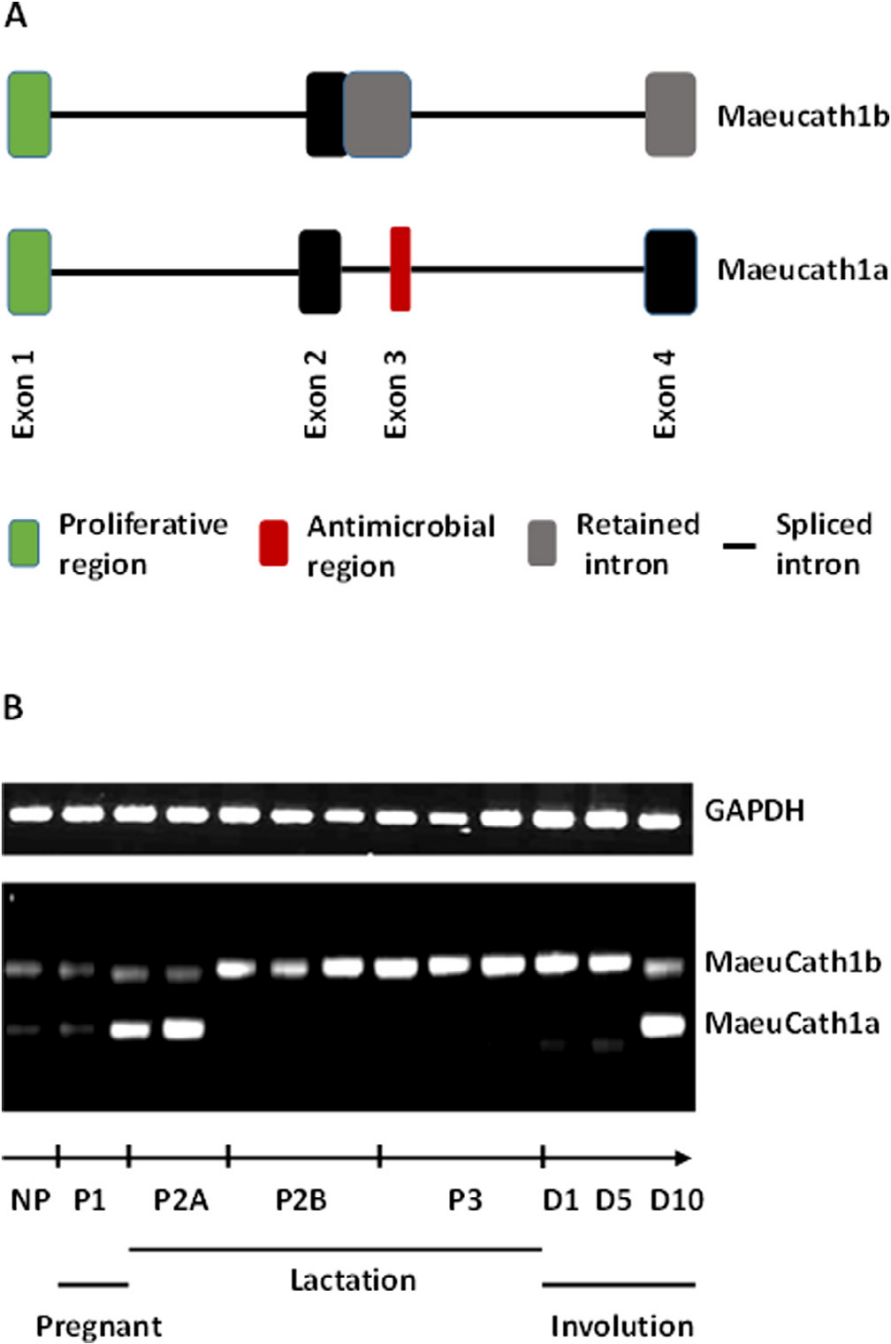
Differential splicing of the *MaeuCath1* gene. (A) The *MeuCath1* gene is differentially spliced and specific transcripts expressed in specific stages of the tammar lactation cycle. (B) PCR analysis of *MeuCath1* expression over the entire lactation period shows Meaucath1a is only expressed during P2A and late involution, while *MeuCath1b* is expressed at low levels during P2A and is upregulated in P2B, P3 and involution. Adapted from (Wanyonyi et al., 2011).

The tammar cathelicidin 1 gene (*MaeuCath1*) revealed two splice variants (Fig. 6; *MaeuCathel1a* and *1b*) that are differentially expressed in the mammary gland throughout the lactation cycle (Wanyonyi et al., 2011). The level of *MaeuCath1a* transcripts increased during early lactation and late involution whereas there was a threefold increase in *MaeuCath1b* expression from P2B lactation until early involution.

The MaeuCath1a protein significantly inhibited a range of bacteria (Wanyonyi et al., 2011). The presence of this protein in milk in the first 48 h *post-partum* and in P2B is consistent with a need to act in synergy with humoral and cellular immune systems to provide protection from pathogens (Daly et al., 2007). *MaeuCath1a* expression at day 10 of involution suggested an additional antibacterial role at a time when the mammary gland is more susceptible to pathogen-mediated mastitis (Oliver and Mitchell, 1983).

#### 8.2.2 A role in proliferation of wallaby mammary epithelial cells

The continued expression of the *MaeuCath1b* splice variant after the timing of immune transfer and the time when the neonate has developed adaptive immunity suggests this protein product may have additional roles for the maintenance and proliferation of mammary epithelia during increasing milk production (Bird et al., 1994, Dove and Cork, 1989). Several studies have suggested a role for cathelicidins in epithelial cell proliferation during wound healing, maintenance and re-establishment of the intestinal barrier integrity and proliferation of lung epithelial cells (Heilborn et al., 2003, Otte et al., 2009, Shaykhiev et al., 2005). Studies reporting WallMEC proliferation in tissue culture models following inclusion of MaeuCath1b in the media confirmed this hypothesis (Wanyonyi et al., 2011).

### 8.3 WAP four-disulphide domain protein-2 (WFDC -2)

**Fig. 7:**
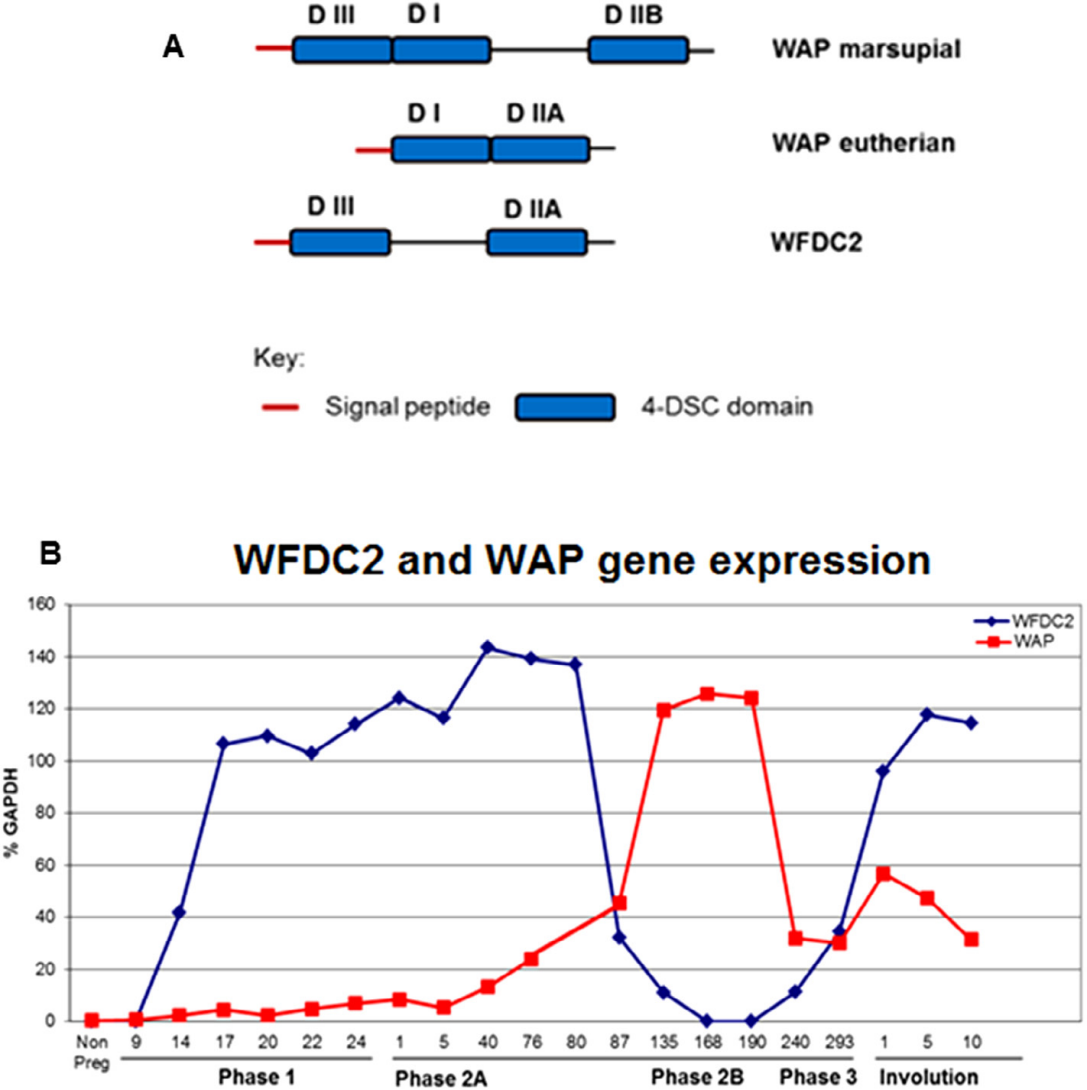
Relationship between WAP and WFDC2. (A) The 4-disulphide domain structure of WFDC2, marsupial WAP and eutherian WAP. Modified from (Sharp et al., 2007a). (B) Expression profiles of the WAP and WFDC2 genes. RNA was extracted from tammar wallaby mammary gland at the indicated stages of pregnancy and lactation. RT-PCR was used to quantify gene expression following normalized expression of the GAPDH gene. Modified from (Watt et al., 2012).

WFDC2 is part of a large family of whey acidic protein (WAP) four disulphide core (DSC) proteins (Fig. 7A; (Bingle et al., 2002, Campbell et al., 1984, Hennighausen and Sippel, 1982, Topcic et al., 2009). Tammar wallaby WFDC2 is comprised of two 4-DSC domains that have previously been annotated domain III on the amino terminal end and domain II at the carboxyl terminal end (Simpson et al., 2000).

WFDC2 gene expression was low in the non pregnant wallaby mammary gland but elevated expression was evident in pregnancy and early lactation (Fig. 7B). The gene was down regulated in mid lactation (P2B) but increased towards the end of P3 and during involution (Watt et al., 2012). These studies by Watt et al. (2012) showed the WFDC2 protein and domain II of the protein had antibacterial activity against *Salmonella enterica*, *Pseudomonas aeruginosa* and *Staphylococcus aureus*. In contrast the WFDC2 protein, domain II and domain III showed no antibacterial activity against *Enterococcus faecalis* suggesting this bioactivity resided within domain II and had strain-specific activity.

The elevated expression of *WFDC2* during pregnancy, early lactation and involution correlates with the timing of increased risk of infection in the mammary gland (Basden et al., 1997, Daly et al., 2007, Old and Deane, 2000) which is largely due to the presence *Staphylococcus aureus*, *Streptococcus spp.* and *Escherichia coli* in the mammary tissue (Barkema et al., 2009, Borm et al., 2006, Bradley and Green, 2001). However, the timing of expression of this gene suggests an additional role in protecting the pouch young during the first 100 days *post-partum* when it is immunocompromised (Basden et al., 1997). It is noteworthy that the antibacterial effect of WFDC2 was directed to potentially pathogenic bacteria and not commensal bacteria (Daly et al., 2007, Old and Deane, 1998, Old and Deane, 2000) and the down regulation of WFDC2 after 100 days *post-partum* when the young detaches from the teat correlated with the development of an immune response in the young.

### 8.4 Whey acidic protein (WAP)

Whey acidic protein (WAP), another member of the WFDC family (Fig. 7A) has been identified in the milk from many eutherian species (Beg et al., 1986, Campbell et al., 1984, Devinoy et al., 1988, Hennighausen and Sippel, 1982, Simpson et al., 1998b) in addition to marsupials (Demmer et al., 2001, Nicholas et al., 2001, Simpson et al., 2000) and monotremes (Sharp et al., 2007b, Teahan et al., 1991). Eutherian WAP has two 4-DSC domains (domain I and II) whereas marsupial WAP has an additional third domain (domain III) (De Leo et al., 2006, Simpson et al., 2000).

Mice, rats and rabbits express the *WAP* gene in the mammary gland during the entire lactation cycle (Campbell et al., 1984, Demmer et al., 2001, Hennighausen and Sippel, 1982) whereas this gene is expressed transiently during mid lactation in the tammar (Fig. 7B; (Simpson et al., 2000) and other marsupials (De Leo et al., 2006, Demmer et al., 2001, Nicholas et al., 2001, Topcic et al., 2009).

Studies using transgenic mice (Burdon et al., 1991) and pigs (Shamay et al., 1992) expressing a mouse *WAP* transgene showed limited mammary development and lactation efficiency and this data was consistent with studies using *in vitro* models (Ikeda et al., 2004, Iwamori et al., 2010, Nukumi et al., 2007, Nukumi et al., 2005). Interestingly WAP gene knock-out mice showed no apparent changes in mammary gland phenotype during the lactation cycle, but the young showed limited development in the later stages of lactation (Triplett et al., 2005).

In contrast earlier studies indicated that the presence of either exogenous tammar WAP or DIII protein alone in culture media specifically increased proliferation of wallaby mammary epithelial cells (Topcic et al., 2009). A protein comprised of DI-II from tammar WAP, which more closely resembles the 2-domain eutherian WAP, had no effect on the proliferation of mammary epithelia cells from the tammar and mice.

Earlier studies have shown that the exogenous expression of *tWAP* and DIII in transfected HC11 and Wall-MEC cells increased cell proliferation and significantly up-regulated the expression of *cyclin D1* and *CDK-4* genes (Topcic et al., 2009) which is consist with a role for tammar WAP as a positive regulator of cell cycle progression of mammary epithelial cells in culture by the regulation of these genes. It is plausible that tammar WAP (and specifically domain III) has a role in the increased DNA synthesis observed in the mammary gland during the mid-phase of lactation (Nicholas, 1988a, Nicholas, 1988b) and subsequent increase in milk production and growth of the young that occurs around the time the young exits the pouch.

These studies suggest DIII of tammar WAP is the functional domain and it is conceivable that evolutionary pressure has adapted the structure and function of the protein and expression pattern of the *WAP* gene with the appearance of the eutherian lineage and accompanying changes in reproductive strategy.

### 8.5 Functional milk-derived peptides

Milk bioactive proteins have a multitude of functions but many of the bioactivities remain inactive until proteolysis releases the latent bioactive peptides (Kanda et al., 2007, Lönnerdal, 2010, Meisel, 2004, Meisel, 2005, Meisel and FitzGerald, 2000, Schmelzer et al., 2007). To examine bioactivity the milk proteins are typically digested by incubating either milk or individual milk proteins with proteases that are found in the gut (pepsin, trypsin and chymotrypsin) (Eriksen et al., 2010, Kitazawa et al., 2007, Picariello et al., 2010, Schmelzer et al., 2007) but it is now apparent that milk has the capacity to process milk proteins with milk-borne proteases. Bioactive milk peptides resulting from proteolytic digestion include functions such as immunomodulatory (Adel-Patient et al., 2012, Qian et al., 2011), antimicrobial (Dallas et al., 2013, Gifford et al., 2005, Liepke et al., 2001), antithrombotic (Chabancea et al., 1995, Fiat et al., 1993), opioid agonists (Gertrud et al., 1985, Meisel and FitzGerald, 2000, Migliore-Samour et al., 1989, Teschemacher et al., 1997), ACE inhibitors (Wu et al., 2013) and proliferative factors (Kanda et al., 2007).

**Fig. 8:**
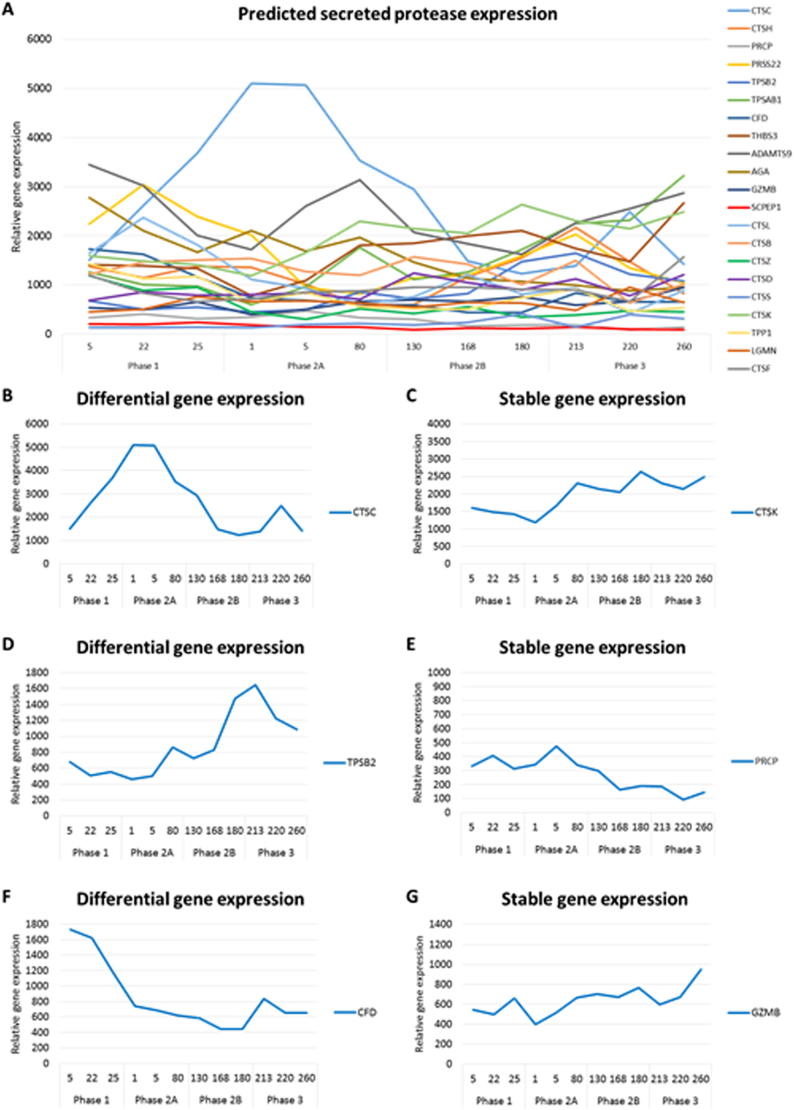
Expression profile of mammary genes coding for secreted proteases expressed during the tammar lactation cycle. (A) Gene expression profiles of proteases were examined by microarray analysis of mammary gland tissue collected from different phases of the tammar wallaby lactation cycle. Examples of some proteases (CTSC, TPSB1, CFD) are differentially expressed in different phases of lactation. (B, D, F). In contrast, the level of expression of some genes (*CTSK, PRCP, GZMB*) remained unchanged during lactation (C,E,G).

These bioactive peptides may be active in the mammary gland to protect the tissue from infection and reduce inflammation during this process, but they may also act in the oral cavity, the gut and potentially move into the peripheral circulation of the suckled young. Therefore it is interesting to explore the potential of the tammar mammary gland to present timed-delivery of these peptides to these environments by the asynchronous activity of both proteases and specific proteins in milk. Gene expression analysis by cDNA sequencing (Lefèvre et al., 2007), microarrays and RNAseq (Lefevre, Sharp and Nicholas, unpublished) of the tammar mammary gland during the lactation cycle has allowed a preliminary examination of some genes coding for secreted proteases. It is apparent that some of these genes are expressed throughout the lactation cycle (Fig. 8), but other genes are expressed at specific phases of the lactation cycle indicating the potential for timed delivery of peptide bioactivity to the mammary gland and the suckled young.

## 9 miRNA

MicroRNAs (miRNAs) are small RNAs that regulate target mRNAs and subsequently influence protein expression levels, thereby having crucial roles in regulating a wide range of cellular functions, such as cell differentiation, proliferation and cell death (Hwang and Mendell, 2006, Song and Tuan, 2006). Recent studies have shown that secretory miRNAs are found in milk (Zhou et al., 2012), saliva (Michael et al., 2010), plasma (Caby et al., 2005) and urine (Pisitkun et al., 2004) suggesting that secretory miRNAs may function in extracellular cell to cell signalling and participate in intercellular regulation of cell function (Kosaka et al., 2013). Milk miRNAs have been reported in several eutherian species including human (Kosaka et al., 2010), bovine (Hata et al., 2010), pig (Gu et al., 2012) goat (Ji et al., 2012) and more recently the tammar (Modepalli et al., 2014). Milk miRNA, are most likely secreted and transported in exosomes (Zhou et al., 2012) to protect the miRNA from degradation although there are studies showing an alternative mechanism using milk fat globules (Munch et al., 2013).

### 9.1 Transport of milk miRNAs in exosomes to the tammar pouch young

Recent studies have shown that the majority of tammar milk miRNA co-purified together with other small RNA in a fraction enriched in exosome-like vesicles (Modepalli et al., 2014). These vesicles were similar to exosomes reported in milk of several eutherian species (Admyre et al., 2007, Gu et al., 2012, Hata et al., 2010, Lasser et al., 2011). These results suggest that miRNAs in tammar milk are likely to be transported through exosome vesicles and potentially play a role in communication of a diversity of potential molecular signals between cells (Creemers et al., 2012). Further analysis of these exosomal miRNAs revealed increased stability under harsh conditions of low pH and high protease activity, indicating that milk miRNAs may successfully be transported into the pouch young digestive system without degradation and survive longer in the gut (Modepalli et al., 2014). Therefore it is likely milk miRNA represent not only potential markers of mammary gland development and activity during the lactation cycle, but also new putative signalling molecules involved in programming development of the suckled young (Kumar et al., 2012).

## 10 Conclusion

The regulation of the lactation cycle in the tammar wallaby has fascinated and challenged scientists for many decades and the interesting interplay between the endocrine, autocrine and paracrine mechanisms that are implicated in this process are now beginning to be better understood. However, it is the timed delivery of bioactives in the milk that play a role in mammary function and protection and development of the suckling young that are of paramount interest. It is ironic that marsupials have long been considered a primitive mammal. The reality is that the mammary gland in these species is very sophisticated in terms of its capacity for temporal delivery of bioactives for multiple targets. Indeed, it appears that the eutherian mammary gland is less sophisticated as many of its previous functions have evolved to be delivered by multiple tissues. It is clear that the marsupial provides a unique opportunity to more easily identify the bioactives that potentially play a role in early development of the fetus.

We have known for some time that significantly premature and low birthweight human babies have acute challenges for survival, largely due to limited development of their lungs and gut. However, it is equally important to note that these babies also have the potential for an increased frequency of mature onset disease in adulthood (Svedenkrans et al., 2013) and this disease status may be more exacerbated if the low birthweight babies have rapidly increased growth rates as part of the procedures to improve their early survival. It appears that significant programming occurs in the earlier stages of development that subsequently impacts on mature onset disease. The tammar wallaby provides a new model to better understand this process of developmental programming. For example, it will be interesting to determine whether the developmental program is set during the 26 day gestation or whether the milk is providing signals to the altricial neonate that have a role in this process. The option of cross fostering neonates to mothers at advanced stages of lactation to exclude the temporal delivery of putative milk bioactives to the young and accelerate growth of the suckled young may shed new light on the process of developmental programming.

## Glossary

Mya: million years ago
Altricial: immature
Bioactive: a molecule that elicits a physiological response
LBW: low birthweight baby
ACL: asynchronous concurrent lactation
P1,2,3: phase 1,2,3 of lactation
MEC: mammary epithelial cells
WallMEC: wallaby mammary epithelial cell
ECM: extracellular matrix
miRNA: a short strand of RNA
